# Crosstalk of ubiquitin system and non-coding RNA in fibrosis

**DOI:** 10.7150/ijbs.93644

**Published:** 2024-07-08

**Authors:** Huamin Zhang, Yutong Zhou, Canhua Jiang, Ni Jian, Jie Wang

**Affiliations:** 1 Department of Immunology, Xiangya School of Medicine, Central South University, Changsha, 410078, China.; 2 Department of Oral and Maxillofacial Surgery, Xiangya Hospital, Central South University, Changsha, 410078, China.

**Keywords:** Fibrosis, Ubiquitination, De-ubiquitination, Non-coding RNA, Epigenetic modifications

## Abstract

Chronic tissue injury triggers changes in the cell type and microenvironment at the site of injury and eventually fibrosis develops. Current research suggests that fibrosis is a highly dynamic and reversible process, which means that human intervention after fibrosis has occurred has the potential to slow down or cure fibrosis. The ubiquitin system regulates the biological functions of specific proteins involved in the development of fibrosis, and researchers have designed small molecule drugs to treat fibrotic diseases on this basis, but their therapeutic effects are still limited. With the development of molecular biology technology, researchers have found that non-coding RNA (ncRNA) can interact with the ubiquitin system to jointly regulate the development of fibrosis. More in-depth explorations of the interaction between ncRNA and ubiquitin system will provide new ideas for the clinical treatment of fibrotic diseases.

## Introduction

Due to the lack of effective treatments and interventions, the overall global prevalence of fibrosis-related diseases is estimated to be approximately 5%, and fibrosis is increasingly recognized as a major health challenge. To date, only two drugs, Nintedanib, and Pirfenidone, which target idiopathic pulmonary fibrosis (IPF), have been approved in several countries for the treatment of fibrotic diseases. Nintedanib has also recently been approved for the treatment of systemic sclerosis-associated interstitial lung disease and progressive fibrotic interstitial lung disease, but these drugs also usually do not stop or reverse disease progression and cause symptoms of nausea, fatigue, and diarrhea in patients due to their non-targeted effects 1. There are still many clinical trials on fibrosis underway, but there is still a long way to go before they are formally operational, and the drug development is still limited, confined to one organ system, and lagging progress. Therefore, an in-depth exploration of the influencing factors affecting the key processes of fibrosis is important for the prevention, diagnosis, and treatment of fibrosis. It has been shown that other epigenetic remodeling, including DNA methylation, ncRNA, and histone modification, plays an important role in the pathogenesis of fibrosis 2. However, the interaction of these epigenetic modalities in fibrosis remains unelucidated. In this review, we summarize the ubiquitin system, non-coding RNAs involved in regulating the key processes of fibrosis, and the interactions between the two in fibrosis, respectively, with the aim of providing new ideas for the development of new drugs for fibrosis.

## Fibrosis

**Figure 1:**
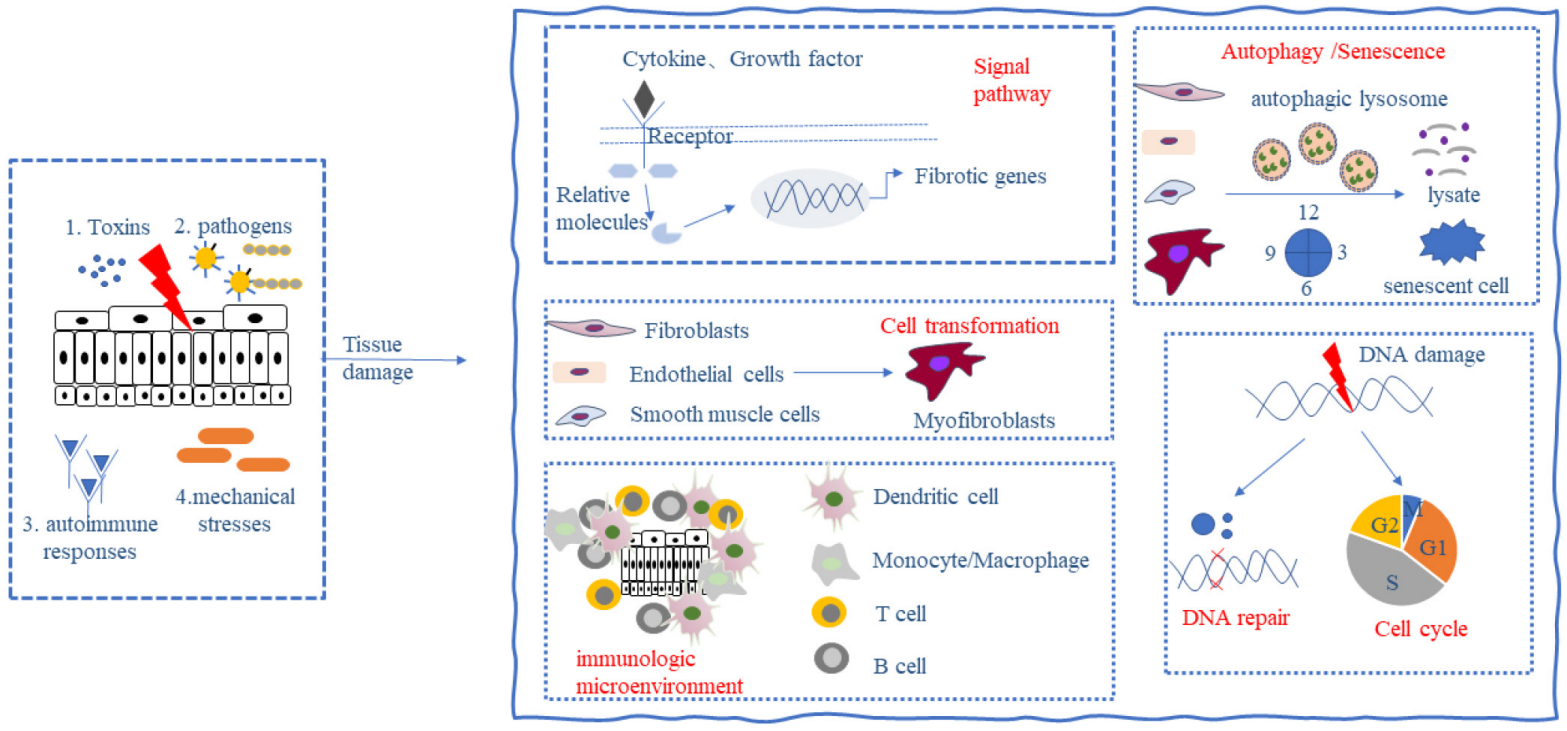
** Key processes in fibrosis formation.** The persistence of toxins, infectious agents, autoimmune responses, and mechanical stresses can cause tissue damage and give rise to the following responses: (1) activation of different signaling pathways; (2) activation and differentiation of fibroblasts and mesenchymal stromal cells; (3) alteration of the immune microenvironment; (4) alteration of cellular autophagy and senescence; and (5) alteration of the cell cycle and DNA damage repair, which ultimately leads to the development of fibrosis.

Fibrosis is the result of a dysregulated tissue repair response during tissue injury, especially chronic inflammatory diseases. Preclinical models and clinical trials have shown that fibrosis is a highly dynamic and reversible process 3, which means that fibrotic tissues and organs may be reversed and cured by human intervention. Primary injury to tissues and organs initiates the fibrotic process, followed by the activation of fibroblasts, immune cells, mesenchymal stromal cells, and other effector cells, leading to the secretion of large quantities of inflammatory mediators and the synthesis of extracellular matrix (ECM) (e.g., collagen and fibronectin). When multiple noxious stimuli including toxins, infectious agents, autoimmune responses, and mechanical stresses persist, ECM components continue to accumulate leading to tissue destruction, organ dysfunction, and even failure 4. Fibrosis occurs in almost all organs, and its formation may involve the following key processes: (1) activation of different signaling pathways; (2) activation and differentiation of fibroblasts and mesenchymal stromal cells; (3) alteration of the immune microenvironment; (4) alteration of cellular autophagy and senescence; and (5) alteration of the cell cycle and repair of DNA damage (Fig. 1). These processes do not occur in chronological order, but most of the time occur at the same time, influence each other strengthen each other, and jointly promote the development of fibrosis.

### Activation of different signaling pathways

TGF-β/smad, WNT/beta-catenin, MAPK/AKT, Notch, EGFR, Hippo, and other signaling pathways are activated in a variety of fibrotic diseases, and a series of cascading reactions promote the progression of fibrosis. The most widely studied is the TGF-β/smad signaling pathway. TGF-β/smad signaling pathway is a recognized fibrotic signaling pathway. It has been found that TGF-β expression is up-regulated in fibrosis of various organs such as the liver, myocardium, and lungs, and activates the TGF-β/smad pathway through the following sequence: TGF-β first binds to the TGFβRII receptor on the cell surface and phosphorylates it, then phosphorylates the adjacent TGFβRⅠ, and the phosphorylated TGFβRⅠ phosphorylates the downstream smad2/3 molecule, which binds to smad4 as a heterotrimer and enters into the nucleus and regulates the transcription of fibrosis-related genes 5, 6. Smad7 is a negative regulator of the TGF-β/smad signaling pathway and competes with smad2/3 to bind to the receptor, preventing the transcription of fibroblast-related genes. MAPK signaling pathway is also one of the pro-fibrotic signaling pathways, e.g., Cai *et al.* found that BMP4 regulates lung fibrosis by inhibiting the ERK/p38 MAPK signaling pathway 7. MAPK, as a mitogenin-activated kinase, can make both serine/threonine and tyrosine phosphorylation, activating four different signaling pathways: ① Ras-Raf-MEK1/2-ERK1/2 ② JNK cascade response ③ P38 cascade response ④ BMK1 (ERK5) cascade response. Several other signaling pathways such as Notch, Hippo, and Wnt/β-catenin have also been reported to be involved in the regulation of the fibrotic process. For example, Yue *et al.* found that specific blockade of the Notch signaling pathway in myofibroblasts upregulated the expression of pro-apoptotic factors Ngfr and Septin4, thus inhibiting the progression of hepatic fibrosis 8; Hippo plays an important role in fibrosis due to its key role in organ development, epithelial homeostasis, tissue regeneration, wound healing, etc. 9. Li *et al.* found that Betulinic acid (an antitumor and antiviral drug) could inhibit bleomycin-induced pulmonary fibrosis by interfering with the Wnt/β-catenin signaling pathway in IPF 10. Of course, these signaling pathways do not work in isolation, and there is a crosstalk between them. For example, activation of the TGF-β/smad signaling pathway can upregulate the expression of PI3K/AKT, CTGF, and WNT, and activation of PI3K/AKT also upregulates the expression of VEGF and FGF, suggesting that the activation of multiple signaling pathways is one of the most important causes of fibrosis. Several existing studies have attempted to alleviate the development of fibrosis by pharmacologically inhibiting the activation of signaling pathways, such as HEC-585, Hydronidone, ZSP1603, and Parsaclisib, which target TGFβRⅠ, p38MAPK, PDGFRs, and PI3K, respectively, and thus modulate the activation of these pathways, and these projects have entered the clinical trial stage, pending the next step of clinical translation.

### Activation and differentiation of fibroblasts and mesenchymal cells

Fibrotic diseases are essentially a consequence of excessive tissue repair response. After tissue injury, the body initiates a repair response in which resting fibroblasts are activated, proliferate, and migrate to the site of injury, secreting extracellular matrix, matrix degradation proteins, etc. to maintain homeostasis. In addition, fibroblasts can differentiate into myofibroblasts with greater collagen synthesis capacity, which can repair damaged tissues by ① expressing α-SMA, ② secreting extracellular matrix/collagen, and ③ secreting integrins/cytokines (VEGF/PDGF/TGF-β) to repair damaged tissues. Normally, myofibroblasts are not found in normal tissues as they transform into low-activity fibroblasts or undergo apoptosis after completing damage repair. However, in sustained tissue injury caused by multiple factors, myofibroblasts can escape apoptosis, proliferate and secrete hydrogen peroxide or angiotensin peptide analogs to induce apoptosis in epithelial cells, and secrete large quantities of cytokines as well as the extracellular matrix, which leads to increased deposition and decreased degradation of cellular matrix, and the gradual progression of organ damage to fibrosis. In most fibrotic diseases, fibroblasts are the main source of myofibroblasts, while in liver fibrosis, myofibroblasts are mainly derived from hepatic stellate cells (HSC). Of course, there are also some mesenchymal cells such as epithelial cells, endothelial cells, adipocytes, and so on can be differentiated into myofibroblasts. Thus, the activation and differentiation of fibroblasts and mesenchymal cells play an important role in fibrotic diseases.

### Alteration of the immune microenvironment

The immune microenvironment is a complex environment composed of cells, molecules, and cytokines inside and outside of fibrotic tissues, which has an important impact on the development and treatment of fibrosis. In the process of fibrosis, tissue injury and parenchymal cell death caused by continuous stimulation by external unfavorable factors will trigger tissue inflammation, and neutrophils, macrophages, T-cells, B-cells, DC cells, etc. will be activated and recruited to the site of injury, and these cells will secrete a variety of cytokines, such as TGF-β, PDGF, IL-1β, IL-6, IL-13, IL-33 and TNF-α, which further change the immune microenvironment of fibrotic tissues and promote fibrosis. M2 macrophages were found to be increased in number in tissues of mouse models and patients with hepatic and pulmonary fibrosis, and fibrotic symptoms were partially alleviated after clodronate liposome removal was used to reduce M2 macrophage expression 11, 12. In IPF, the Treg/Th17 ratio is dysregulated and the number of Tregs is upregulated, and bleomycin-induced pulmonary fibrosis is exacerbated in mice following Treg removal using CD25 antibody 13; depletion of Tregs *in vivo* also exacerbates hepatic fibrosis and dermal fibrosis in bile duct ligated mice 14, 15. Immune cell disorders accompanied by cytokine disorders have also been demonstrated in a variety of fibrosis. For example, in oral submucous fibrosis, the expression of several cytokines, such as TGF-β, CTGF, PDGF-BB, and IL-17A, is elevated and strongly correlated with the disease process; in IPF, several growth factors, chemokines, and interleukins are dysregulated, and researchers have designed cytokine-targeted drugs based on these phenomena and have entered the phase of clinical trials, which have developed new options for the treatment of fibrosis.

### Alterations in cellular autophagy, senescence

Autophagy is a process in eukaryotic cells in which lysosomes are involved in the degradation of long-lived proteins, damaged organelles, and invading microorganisms to maintain intracellular homeostasis, and the entire process of autophagy occurs is mediated by autophagy-related genes (Atg) and their encoded family of Atg protein molecules, over 30 genes including LC3, Beclin1, PI3K. The selective loss of endothelial autophagy during liver injury *in vivo* was found to result in cellular dysfunction and reduced intrahepatic nitric oxide by Ruart *et al.* Loss of autophagy also impairs the ability of hepatic endothelial cells (LSECs) to deal with oxidative stress and exacerbates fibrosis 16. Cell aging (Cell aging) refers to the process of change in which the cell's ability to proliferate and differentiate and its physiological functions gradually decline over time as the cell performs its life activities. Abnormal senescence of normal cells can lead to the occurrence of diseases. Senescent cells stagnate in growth and are unable to proliferate, but they remain metabolically active and can secrete "senescence-associated secretory phenotypes" that lead to the development of pathological conditions. Ovarian fibrosis is a disease associated with oocyte senescence 17, and pulmonary fibrosis has also been treated by anti-aging 18. Yao *et al.* found that senescence of alveolar type 2 (AT2) cells is sufficient to drive progressive pulmonary fibrosis. Early attenuation of senescence-associated pathways and elimination of senescent cells are effective treatments to prevent pulmonary fibrosis 19. Cellular autophagy and senescence promote the development of fibrosis to some extent.

### Alterations in the cell cycle, DNA damage repair

It is well known that DNA stores the genetic information on which organisms depend for survival and reproduction, so maintaining the integrity of the DNA molecule is crucial for cells. Both the external environment and internal factors of organisms can lead to damage or alteration of DNA molecules, and if DNA damage or alteration of genetic information cannot be corrected, it will have unimaginable consequences on the function and survival of the organism. Shen *et al.* found that prolonged exposure to microplastics induces DNA damage in the nucleus and mitochondria and that the double-stranded DNA (dsDNA) fragments are translocated into the cytoplasm and triggers the DNA-sensing aptamer STING, which in turn activates the cGAS/STING pathway and initiated a downstream cascade of reactions that translocated NF-κB to the nucleus, upregulated the expression of pro-inflammatory cytokines, and thus ultimately promoted liver fibrosis 20. This is why the ability to repair DNA damage acquired by biological cells during evolution is so important. The cell cycle is the process that occurs from the beginning of one cell division to form a daughter cell until the next cell division to form a daughter cell. Precise regulation of the cell cycle is essential for tissue homeostasis and development, and cell cycle dysregulation has been associated with many human diseases, such as damage caused by toxins, hypoxia, and metabolic disorders that can stimulate renal cell cycle arrest or hyperproliferation, which is closely associated with renal fibrosis 21. It is thus clear that alterations in the cell cycle, and DNA damage repair are also one of the key processes in fibrosis.

## The ubiquitin system regulates the development of fibrosis

### Ubiquitin system

#### Ubiquitination

Post-translational modification (PTM) of proteins is a sensitive, rapid, and reversible way to regulate cellular homeostasis, including methylation, acetylation, glycosylation, phosphorylation, and ubiquitination. Compared with other post-translational modifications of proteins, ubiquitination, as a class of protein modifications with more complex modes of action and more diverse outcomes, plays an important role in physiological and pathological processes 22. Ubiquitination is accomplished by the synergistic action of ubiquitin proteins and ubiquitinases. Ubiquitin protein, a small molecular weight protein, is present in all eukaryotes and consists of 76 amino acids. The glycine at the C-terminus of the activated ubiquitin molecule covalently binds to the binding site of the substrate protein, causing the substrate protein to be modified by ubiquitination. Ubiquitinases include ubiquitin-activating enzyme (E1), ubiquitin-conjugating enzyme (E2), and ubiquitin ligase (E3). Two E1 enzymes, 40 E2 enzymes, and more than 600 E3 enzymes are known (Table 1). Among them, E3 ubiquitin ligases can specifically recognize the substrate, and the known E3 ligases can be classified into HECT, RING, and U-box domains according to their structural domains. The HECT structural domain can form a thioester bond with ubiquitin and directly catalyze the ubiquitination of substrate proteins, whereas the RING structural domain transfers activated ubiquitin molecules to substrate proteins, which in turn catalyzes substrate ubiquitination, and the U-box structural domain may recognize unfolded and misfolded proteins through its role in interacting with molecular chaperones HSC/HSP70, i.e. The E1 enzyme, when ATP-supplied, binds to a cysteine residue (Cys) in the tail of the ubiquitin molecule, mutating the Cys to an alanine (Ala), forming an E1-Ub thioester intermediate that activates the ubiquitin molecule, then E1 transfers the activated ubiquitin molecule to E2 and forms E2-Ub thioester intermediate, E2 and some different kinds of E3 enzymes recognize the target protein, E3 transfers the ubiquitin molecule to the substrate molecule, the carboxylate group of G67 at the carbon end of the ubiquitin molecule forms an isopeptide bond with the amino group of lysine on the substrate protein, and the amino acid sites of ubiquitin molecule, such as Met1, Lys6, Lys11, Lys27, Lys29, Lys33, Lys48, and Lys63, undergo mono-, poly-, or poly-ubiquitination, and the substrate can be ubiquitinated 23.

#### Deubiquitination

Deubiquitination is the process of removing ubiquitin from the substrate and recycling ubiquitin in the cytoplasmic pool in the presence of deubiquitinating enzymes. Deubiquitinating enzymes (DUBS), more than 100 DUBS are known, including ubiquitin-specific proteases (USPs), ovarian tumor-associated proteases (OTUs), and monocyte chemotaxis-inducing proteins (Table 1). After ubiquitination is completed, DUBS can act on the protein substrate after ubiquitination modification, severing the linkages between the ubiquitin chain and the protein substrate as well as between the ubiquitinated chains, so that the ubiquitin molecule is detached from the ubiquitinated protein substrate or the proteasome, and re-enters the intracellular protein cycle 24.

Ubiquitination and deubiquitination of proteins can be involved in the regulation of various physiopathological processes such as immune response, mitochondrial autophagy, DNA damage repair, cell cycle, epigenetic inheritance, apoptosis, and protein degradation, which can in turn regulate the growth and development of organisms as well as the progression of diseases. For example, in the immune response, the inflammatory vesicle NLRP3 25, type I interferon 26, and the nuclear transcription factor NF-κB 27 can be directly or indirectly affected by ubiquitination to regulate the immune response; whereas in mitochondrial autophagy, ubiquitination of PINK1 and Parkin is a key event 28. However, the most common role of ubiquitination is still protein degradation, where ubiquitinated protein molecules are degraded to small polypeptides in a 26S protein molecule. Numerous studies have now shown that the ubiquitin system plays a pivotal role in fibrotic diseases (Table 2).

### The ubiquitin system regulates the development of fibrosis

#### The ubiquitin system regulates the activation of different fibrosis-related signaling pathways

Current studies have found that the ubiquitin system can directly or indirectly regulate many signaling pathways that are closely related to the development of fibrosis, including TGF-β/smad, WNT/beta-catenin, MAPK/AKT, Notch, EGFR, Hippo, etc. Researchers have found that the ubiquitin system regulates the entire TGF-β/smad signaling pathway. The E3 ubiquitin ligase Fbxw7 inhibits TGF-β production by interacting with the transcription factor c-Jun and mediating the ubiquitination degradation of its K48 linkage 53. The deubiquitinating enzyme USP4 upregulates the expression of TGFβRI by deubiquitinating it and the ability to respond to TGF-β signaling 56. The transcription factor RUNX1 binds to the promoter of the ubiquitin-specific protease USP9X and promotes the expression of USP9X, which binds to smad1 and stabilizes smad1 expression 57. The E3 ubiquitin ligase WWP2 interacts with smad2 to promote its mono-ubiquitination regulates the nucleocytoplasmic shuttling and transcriptional activity of smad2 and mediates TGF-β induced pro-fibrotic activity 46. Zinc finger (POZ) protein (SPOP) is a substrate-recognizing adaptor of the Cullin-3 (CUL3)/RING-type E3 ligase complex, where SPOP binds to the activated protein C kinase 1 receptor (RACK1) and induces its ubiquitination and degradation by recognizing Ser/Thr-rich motifs on RACK1, leading to smad3 activation 58. The ubiquitin-like protein FAT10 can directly bind to the K378 site of smad3 through its C-terminal glycine residue, resulting in the degradation of smad3 37. TGF-β and oxidative stress can collaborate to drive the progression of fibrosis by the mechanism that the NADPH oxidase DUOX1 can inhibit the interaction between phosphorylated smad and the ubiquitin ligase NEDD4L, preventing NEDD4L-mediated ubiquitination and degradation of p-smad3 59. The interaction between FMO2 (flavin-containing monooxygenase 2) and CYP2J3 (cytochrome p450 superfamily 2J3) disrupts the interaction between CYP2J3 and Smurf2 (smad-specific E3 ubiquitin ligase 2) in the cytoplasm, leading to the translocation of Smurf2 from the cytoplasm to the nucleus, thereby inhibiting smad2/3 signaling 60. Up-regulation of the heat shock protein HSP47 recruits the ubiquitin-specific protease USP10, which deubiquitinates smad4 and activates the smad4 pathway 61. USP25 interacts with smad4 and reduces polyubiquitination of the smad4 K63 site, which inhibits the nuclear translocation of smad2, thereby inhibiting activation of the TGF-β/smad pathway 62. Elevated expression of the calcium-sensing receptor CasR upregulates the expression of Smurf2, leading to increased levels of ubiquitination of smad7, which attenuates its inhibitory effect 63. Ubiquitination of smad7 by Smurf2 to degrade smad7 has also been demonstrated in dermal fibrosis 64. Elevation of CasR concomitantly upregulates the expression of the E3 ubiquitin ligase Itch, which can interact with smad7 to ubiquitinate it for degradation, and upregulates the phosphorylation levels of smad2 and smad3, activating the TGF-β/smad pathway 52. Potentially TGF-β can inhibit Arkadia-mediated ubiquitination degradation of smad7 and promote activation of the TGF-β/smad signaling pathway 65. The E3 ubiquitin ligase, KLHL42, interacts with the phosphatase 2 regulatory subunit 5ε (PPP2R5ε), accelerating the degradation of PPP2R5ε, and the reduced expression of PPP2R5ɛ directly results in more phosphorylation of PP2A substrates, activating the PP2A pathway and indirectly accelerating TGF-β signaling 66. USP11 binds to the epidermal growth factor receptor (EGFR) and deubiquitinates and protects the EGFR from proteasome-dependent degradation, enhancing the transduction of the downstream signaling pathway of TGF-β 67.

The ubiquitin system can modulate key molecules of the MAPK signaling pathway and thereby regulate the fibrosis process. The E3 ligase TRIM31 interacts with the polyubiquitination of the K48 linkage of the lysine at position 72 on MA3PK7 and catalyzes the degradation of MA3PK7, which directly regulates the activation of MAPK signaling 55. The E3 ubiquitin ligase TRIM38 interacts with and directionally degrades the TAK1-binding proteins TAB2 and TAB3, inhibiting the phosphorylation of transforming growth factor-β-activated kinase 1 (TAK1), which serves as an upstream kinase of the MAPK pathway, and the reduction of its phosphorylation indirectly inhibits the MAPK signaling pathway 68. Miao *et al.* also found that the up-regulation of ubiquitin-specific protease USP19 directly abolished the TAK1-induced P38/ JNK1/2 signaling 69. In contrast, Yuan *et al.* found that the E3 ligase RNF207 triggers autophosphorylation of TAK1 and activation of the downstream p38 and c-Jun N-terminal kinase (JNK) 1/2 signaling pathways by facilitating ubiquitination of the K63 linkage to TAB1 70. Increased levels of the E3 ligase CBL lead to increased ubiquitination, internalization, and degradation of PDGFR, which negatively regulates MAPK and AKT downstream signaling 71. The E3 ubiquitin ligase HUWEI binds to EGFR promotes ubiquitination and degradation of EGFR, inhibits EGF binding to EGFR, and inhibits activation of downstream MAPK signaling pathways 72. The deubiquitinating enzyme ubiquitin aldehyde binding 1 (OTUB1) directly binds to tumor necrosis factor receptor-associated factor 6 (TRAF6) and inhibits its lysine 63-linked polyubiquitination, thereby inhibiting the activation of ASK1 and its downstream MAPK pathway 73.

The ubiquitin system also plays an important role in the activation of other pathways. Delta-like ligand 1 (DLL1) is a transmembrane ligand for Notch1, Notch2, and Notch3 receptors, and FBXW7 interacts with DLL1 to degrade DLL1, which blocks the transduction of the Notch signaling pathway 54. USP10 directly binds to NICD1 (Notch1 receptor), enabling stable expression of NICD1 and regulating activation of the Notch signaling pathway 74. MOB1 acetylation promotes activation of the Hippo signaling pathway, and the E3 ligase praja2 interacts with and ubiquitinates degradation of MOB1 to inhibit Hippo signaling 75. Deletion of the ubiquitin ligase BRCA1-associated protein (BRAP) inhibits Hippo pathway signaling 76. The deubiquitinating enzyme USP2 interacts with β-catenin to deubiquitinate β-catenin without degradation, which in turn activates Wnt/β-catenin signaling 29. NEDD4L can also directly interact with β-catenin to degrade β-catenin while inhibiting the activation of β-catenin signaling 49.

Multiple signaling pathways can ultimately activate the NF-κB pathway, allowing the transcription factor NF-κB to enter the nucleus to regulate the expression of fibrosis-related genes such as α-SMA and COL-1. Researchers found that NF-κB can bind to the promoter of ubiquitin carboxy-terminal hydrolase 1 (UCHL1) to promote its transcription, and UCHL1 in turn activates the NF-κB pathway, forming positive feedback 41. The deubiquitinase OTULIN negatively regulates polyubiquitin signaling from methionine-1 (M1) by removing chains conjugated by LUBAC, modulating the activation of the NF-κB pathway 77.

#### The ubiquitin system regulates the activation and differentiation of fibroblasts and mesenchymal cells

Investigators demonstrate that the reduction of ubiquitin E3 ligase NEDD4L in myofibroblasts accelerates pulmonary fibrosis 48, and inhibition of UCHL1 (ubiquitin carboxy-terminal hydrolase 1) expression attenuated myocardial fibrosis 39. Therefore, exploring the regulation of activation and differentiation of fibroblasts and mesenchymal stromal cells by the ubiquitin system is crucial for the prevention and treatment of fibrotic diseases. Xu *et al.* found 29 that the expression of the deubiquitinating enzyme USP2 was elevated in angiotensin II-induced cardiac fibroblasts, and that its interaction with β-catenin prevented β-catenin deubiquitination from being degraded, thereby promoting the activation, proliferation, and collagen synthesis of cardiac fibroblasts. Cheng *et al.* found that the possible mechanism of fibroblast activation due to TGF-β treatment was that TGF-β treatment upregulated the expression of UHRF1 (ubiquitin-like enzyme 1 containing PHD and RING structural domains), which induced methylation of the beclin1 promoter and inhibited the expression of beclin1, thus leading to fibroblast activation 78. It has been shown that GJA1 mRNA, which translates the gap junction protein connexin 43 (Cx43) during epithelial-mesenchymal transition (EMT), initiates internal translation and produces a deleted version of Cx43 to regulate gap junctions. Sun *et al.* found that the deubiquitinating enzyme USP9X deubiquitinates Cx43 to inhibit the process of high glucose-induced EMT, which in turn inhibits renal tubulointerstitial fibrosis 79. Kim *et al.* 80 found that tetratricopeptide repeats domain 3 (TTC3), a ubiquitin E3 ligase, promotes TGF-β1-induced EMT and myofibroblast differentiation by inducing the ubiquitination and proteasomal degradation of smurf2. Mutation of the start codon of the ubiquitin ligase KLHL24 results in the formation of a gain-of-function mutant, KLHL24-DeltaN28, which mediates excessive degradation of vimentin, failing to maintain effective proliferation and activation of fibroblasts during wound healing, which in turn prevents activation of myofibroblasts and thus attenuates bleomycin-induced skin fibrosis 81. Zhou *et al.* found that the antifibrotic drug Celastrol could enhance the interaction between CAND1 and Culin1, promote the activity of SKP1/Culin1/F-box ubiquitin ligase, and inhibit the differentiation of lung fibroblasts, thus exerting an antifibrotic effect 82. Neuropilin-1 (NRP-1) promotes the abnormal deposition in hepatic fibrosis, and the expression of NRP-1 is significantly elevated in hepatic fibrotic liver tissues. Zhao *et al.* found that USP9X is a key deubiquitylating enzyme for the stabilization and high activity of NRP-1, and that USP9X binds to and stabilizes the expression of NRP-1, and that up-regulated NRP-1 can activate hepatic stellate cells to promote the progression of liver fibrosis 83.

#### The ubiquitin system regulates the immune microenvironment during fibrosis

Researchers have found that the ubiquitin system also regulates the immune microenvironment during fibrosis 84. The E3 ligase WWP2 interacts with the transcription factor IRF7 to promote its non-degradative mono-ubiquitination, which in turn regulates monocyte/macrophage infiltration as well as polarization in myocardial tissues, and promotes the secretion of CCL5, which alters the immune microenvironment of the cardiac tissue 44. Ubiquitin-specific peptidase 1 (USP1) interacts with SNAIL to deubiquitinate it and stabilize its expression, which in turn increases the expression of CXCL1 in liver tissues and promotes hepatic fibrosis 85. USP38 interacts with IL-33R to deregulate the polyubiquitination of its K27 linkage at K511, whereas the E3 ubiquitin ligase tumor necrosis factor receptor-related factor 6 (TRAF6) catalyzes the polyubiquitination of IL-33R here, and together they regulate IL-33R levels and signaling, which in turn modulates the local inflammatory response to lung fibrosis 86. The expression of E3 ubiquitin ligase MIDI can be upregulated by tumor necrosis factor-related apoptosis-inducing ligands (TRAIL), which inactivates PP2A (protein phosphatase 2A) ubiquitination and promotes lung fibrosis 87. Serine/threonine protein kinase 6 (AuRKA) interacts with heat shock protein 90 (HSP90), whereas HSP90 inhibitors promote ubiquitin-dependent degradation of AuRKA, and a reduction in AuRKA expression induces the differentiation of immature macrophages and reduces the mutational burden of primary myelofibrosis 88. Ubiquitin editing enzyme 20 (A20) interacts with and inactivates protein kinase glycogen synthase-3β (GSK-3β) and inhibits the degradation of the transcription factors CCAAT and enhancer-binding protein β (C/EBPβ) within alveolar macrophages (AMS), leading to a compliant phenotype of AMS, i.e., the M2 phenotype promotes the pulmonary fibrosis (PF) development 34. Interference with GSK-3β/OTUA (deubiquitinating enzyme) interaction in macrophages accelerates the degradation of the transcription factor CBP and attenuates pulmonary fibrosis 89. The glucocorticoid SGK1 phosphorylates NEDD4L, which prevents NEDD4L from performing its ubiquitination function, which in turn induces the differentiation of Th cells into Th17 and Th2 phenotypes, as well as hinders Treg development and promotes inflammatory fibrosis 90. Tong *et al.* found that ubiquitin ligases CBL and CBL-B mutations enhanced FLT3-mTOR signaling by the mechanism that CBL and CBL-B mutations caused them to lose the function of ubiquitinating degradation of FLT3, and the upregulation of FLT3 expression enhanced FLT3-mTOR signaling, allowing more CD8 alpha(+)/CD103(+) DCs (cDC1s) to infiltrate into liver tissues, secrete large amounts of inflammatory factors and chemokines, and exacerbate hepatic fibrosis 91. Tong *et al.* found that the E3 ubiquitin ligase RNFT2 (RING refers to transmembrane domain-containing protein 2, also known as TMEM118), which can interact with IL-3Rα and cause it to undergo ubiquitination and degradation, with reduced expression of IL-3Rα and diminished responsiveness to IL-3, and the biological effects of IL-3 in amplifying pro-inflammatory signaling and cytokine storms are greatly diminished, which, in turn, attenuates the occurrence of fibrosis 92.

#### The ubiquitin system regulates fibrosis-associated cell autophagy, senescence

The ubiquitin system regulates cellular autophagy and senescence and then modulates fibrosis. The ubiquitin-specific protease USP13 interacts with and deubiquitinates beclin1, increasing cellular autophagic activity and inhibiting age-associated lung fibrosis 31. The E3 ubiquitin ligase TRIM44 binds to the K48 ubiquitin chain on proteins and promotes aggregated protein clearance while also facilitating the activation of autophagy by promoting the oligomerization of SQSTM1/p62, thereby rapidly increasing the rate of aggregated protein clearance 93. The E3 ubiquitin ligase TRIM65 can inhibit mitochondria-dependent apoptosis and autophagy by modulating Jak1 ubiquitination, ultimately attenuating isoproterenol (ISO)-induced cardiac hypertrophy 94. The multisubunit E3 ubiquitin ligase anaphase-promoting complex/cyclosome (APC/C) coactivator Cdh1 stimulates the ubiquitination degradation of SIRT1, a member of the mammalian sirtuin family of nicotinamide adenine dinucleotide (NAD)-dependent deacetylases, and regulates cellular senescence 95.

#### The ubiquitin system regulates DNA repair processes and cell cycle progression in fibrosis-associated cells

The ubiquitin system regulates cell cycle progression and DNA repair processes and thus fibrosis. E3 ubiquitin ligase WWP2 interacts with the BRCT structural domain of poly (ADP-ribose) polymerase (PARP1) and mediates PARP1 degradation through the ubiquitin-proteasome system, which in turn regulates DNA repair processes 45. The binding of the E3 ubiquitin ligase FBW7 and telomere-protective Protein 1 (TPP1) promotes multisite polyubiquitination of TPP1 and accelerates TPP1 degradation, which triggers telomere uncapping and DNA damage response and mediates tissue fibrosis 96. The ubiquitin-specific protein USP7 interacts with and stabilizes the expression of human leukocyte antigen-F-adjacent transcript 10 (FAT-10), which mediates the up-regulation of CHK1, thereby promoting CHK1-mediated G2/M arrest in renal tubular mesenchymal-epithelial cells and contributing to renal fibrosis 36. Alcohol-mediated down-regulation of dual-specificity phosphatase-1 (DUSP1) interrupts the interaction of DUSP1/Cullin-1 (CUL1), a member of the SKP1-Cullin 1-F-box protein (SCF) E3 ubiquitin-protein ligase complex, which has been identified as a ubiquitylation of parkin substrate. A well-recognized role of ubiquitinated Culin1 is to promote the activation of Cyclin-E and thereby regulate the cell cycle 97. The E3 ubiquitin ligase, parkin, is a key regulator of mitosis, regulating the cell cycle and, in turn, cell proliferation 98.

All the above studies show that the ubiquitin system is involved in the key process of fibrogenesis and development and is a potential therapeutic target for fibrosis (Figure 1). Drugs targeting the ubiquitin-proteasome system have shown good therapeutic effects in the treatment of cancer and inflammatory diseases. For example, the first-generation proteasome inhibitor Bortezomib and the second-generation proteasome inhibitors such as Carfilzomib and Ixazomib are used for the treatment of multiple myeloma, a hematological tumor, and the immunoproteasome inhibitor ONX-0914 has shown good therapeutic effects in the treatment of systemic lupus erythematosus, rheumatoid arthritis, and other inflammatory diseases with increased immunoproteasomes. In addition, drugs targeting deubiquitinating enzymes such as the deubiquitinating enzyme Rpn11 inhibitor Capzinmin have been shown to inhibit the proliferation of several tumor cell lines 99. Although drugs targeting the ubiquitin system for the treatment of fibrosis have not yet appeared in the clinic, Panyain *et al.* found that potent and selective UCHL1 inhibitors blocked the pro-fibrotic response in a cellular model of IPF, which supports the potential of UCHL1 as a potential therapeutic target for fibrotic diseases 100, and fibrotic diseases are also a type of inflammatory disease, and the development of therapeutic drugs targeting the ubiquitin system in the future has been comparatively successful. There is a solid foundation for the future development of therapeutic agents targeting the ubiquitin system.

However, therapeutic drugs targeting the ubiquitin system have some drawbacks, such as poor effect on solid tissues, resistance of the body to long-term use, and dose toxicity. Therefore, more research is needed to address these issues. Researchers have studied deeply and found that non-coding RNA (non-coding RNA, ncRNA) can regulate the expression of the ubiquitin system. For example, F-box proteins (FBPs) are proteins containing the F-box structural domain, one of the three subunits of the SKP1-Cullinl-F-box protein (SCF) E3 ligase complex. FBPs perform their biological functions by ubiquitination and degradation of downstream substrates. Multiple E3 ubiquitin ligases of the FBPs family such as FBXW7, FBXW8, β-TrcP, FBXW11, Skp2, etc., have been reported to be regulated by ncRNAs, such as microRNAs (miRNAs) miR-27 101, miR-770 102, miR-182 103; long non-coding RNAs (lncRNAs) MTIJP 104, CASC2 105; and circular RNA (circRNA) circFBXW7 106 have all been reported to regulate FBXW7 expression. This implies that the regulatory role of non-coding RNAs needs to be taken into account when targeting the ubiquitin system as a therapeutic target for fibrosis and implies that non-coding RNAs may work together with the ubiquitin system to regulate the progression of fibrosis.

## Advances in ncRNA regulation of fibrosis

### Overview of ncRNA

About 90% of genes in eukaryotic genomes are transcribed genes. However, only 1-2% of these transcribed genes encode proteins, while the rest are transcribed as ncRNAs because they lack open reading frames long enough to encode proteins. ncRNAs are divided into two main types: basic structural and regulatory ncRNAs. Basic structural ncRNAs, such as ribosomal RNAs, transfer RNAs, and mini-nucleosomal RNAs, appear to play a role similar to that of housekeeping genes in translation and splicing. Regulatory ncRNAs include miRNAs, lncRNAs, and circRNAs, and most of the research nowadays focuses on regulatory ncRNAs.

miRNA, an endogenous non-coding single-stranded RNA of 18-24 nucleotides in length, is currently the most thoroughly studied ncRNA. Mature miRNAs exert their functions by forming a silencing complex (RISC), which includes proteins such as Dicer and Ago2 that bind to miRNAs. miRNAs directly degrade or inhibit target mRNAs by pairing either completely or incompletely with the target mRNA's 3'-UTR, thereby regulating post-transcriptional gene expression and participating in a variety of physiological and pathological processes 107, 108.

LncRNAs are a class of endogenous noncoding single-stranded RNAs that are more than 200 nucleotides in length. LncRNAs can be categorized based on their location on the genome and their location relative to protein-coding genes, and are classified as intronic lncRNAs, intergenic regions of lncRNAs, antisense lncRNAs, heterogeneous lncRNAs, and enhancers of lncRNAs. These lncRNAs can bind to DNA, RNA, and protein. When binding to DNA, they can directly regulate gene transcription by binding to gene promoters; when binding to RNA, the most common way is to act as ceRNA, targeting miRNAs and inhibiting their function and thus exerting their biological functions; they can also bind to mRNAs to regulate gene expression at the post-transcriptional level. LncRNAs are also bound to many types of proteins, such as transcription factors, enzymes, etc. They can act as protein decoys to directly regulate the activation and silencing of proteins, or as protein scaffolds to facilitate the interaction between two proteins 109, 110. In recent years, due to the development of proteomics and ribosome mapping technologies, a small number of lncRNAs have been translated into small molecule polypeptides and thus perform biological functions by ① Cap-dependent, ② IRES-dependent, ③ m6A-dependent, and ④ sORF-dependent ways 111.

CircRNA, an endogenous single-stranded circular non-coding RNA of several hundred to several thousand nucleotides in length, can be classified into three categories according to its components: exonic circular RNA (EcRNA), intronic circular RNA (CiRNA), and exon-intronic circular RNA (EliRNA). Unlike other types of non-coding RNAs, the parent gene of circRNAs undergoes reverse splicing during maturation, with the 5' and 3' ends joined in a covalently closed loop. circRNAs perform their functions in a manner similar to lncRNAs, however, lncRNA has a methyl guanosine cap (m7G) at the 5' end and a polyadenylate (poly) tail at the 3' end, and thus is easily degraded, whereas circRNA deficient in two free ends, so they can resist the degradation effect of nucleic acid exonucleases, thus having a longer half-life and higher stability 112. This implies that circRNAs have better prospects for clinical applications.

Several studies have now shown that changes in ncRNA expression can be used as diagnostic indicators and therapeutic targets for a variety of cancers. For example, the use of changes in the expression level of miR-10b for the early diagnosis of glioblastoma (NCT01849952) and the use of high expression of lncRNA H19 in tumors, with the help of its promoter to initiate PEI-imported BC-819 (expressing diphtheria toxin A) for the treatment of non-muscle invasive bladder cancer (NCT03719300) have entered the clinical trial stage. This shows that ncRNAs have the potential to become diagnostic indicators and therapeutic targets for a variety of diseases, including fibrosis. The regulatory role played by ncRNAs in human physiopathological processes should not be ignored.

### ncRNA regulates the development of fibrosis

#### ncRNA regulates the activation of different fibrosis-related signaling pathways

Studies have shown that non-coding RNAs can modulate the activation of multiple signaling pathways affecting the progression of fibrosis. The classic signaling pathway of fibrosis, the TGF-β/smad pathway, remains the most thoroughly investigated pathway. Almost all individual molecules of the TGF-β/smad pathway can be regulated by ncRNAs, e.g., circHIPK3, miR-29b can target TGF-β; miR-let-7i-5p, lncSNHG20 target TGFβRⅠ; miR-30c, miR-9-5p target TGFβRII; miR-27a-3p target smad2; miR-145, miR-370, lncRNA CRNDE target smad3; miR-146a, miR-27-3p target smad4; miR-877-3p, circHNRNPH1 target smad7, which all suggest that ncRNAs play an important role in TGF-β/smad activation. In addition, ncRNAs can target key molecules of other signaling pathways. For example, miR-718, miR-21, miR-338-3p can directly target PTEN, a key molecule in the PI3K/AKT signaling pathway; miR-21, miR-32-5p can target PDCD4 and DUSP1 to regulate the activity of the MAPK signaling pathway, respectively; and miR-33a-3p and miR-154 target DKK to regulate Wnt/ β-catenin signaling pathway. Thus, it is evident that non-coding RNAs play an integral role in fibrosis-related signaling pathways 113-115.

#### ncRNA regulates activation and differentiation of fibroblasts and mesenchymal cells

In PF, miR-22 116 inhibited lung fibroblast differentiation. miR-375 117 prevented lung fibroblast differentiation by blocking the P38 mitogen-activated protein kinase (P38) pathway; miR-424 118, 119 not only targeted Slit2 to promote lung fibroblast differentiation, but also promotes epithelial EMT. In systemic sclerosis, miR-16-5p 120 inhibits myofibroblast cell activity; miR-30c 121 targets alpha 2AP to inhibit fibroblast differentiation. In oral mucosal fibrosis, lncRNA FENDRR 122 could directly bind to miR-214 and regulate betaine-induced reactive oxygen species accumulation and myofibroblast differentiation; miR-424 123 could target TGIF2 to inhibit myofibroblast cell activity; miR-155 124 could target RPTOR to increase myofibroblast cell activity; betaine could promote miR-21 expression in oral mucosal fibroblasts, and miR-21 promotes oral mucosal fibroblast activation 125; miR-29c 126 targets TPM1 to inhibit myofibroblast activity; and twist up-regulates miR-10b expression to promote oral mucosal myofibroblast activation 127. In pathological scarring diseases, miR-181a 128 can target SIRT1 to promote fibroblast differentiation; miR-145 129 can target KLF4 to induce fibroblast differentiation. In myocardial fibrosis, miR-574-5p 130 targets ARID3A to regulate cardiac fibroblast differentiation; miR-21 131 can polarize macrophages toward M1 and mainly determines communication between macrophages and fibroblasts, promoting the differentiation of quiescent fibroblasts to myofibroblasts; and cardiomyocyte-derived exosome miR-92a can be used in mediate post-ischemic myofibroblast activation *in vitro* and *ex vivo* 132; lncRNA TUG1 133 regulates cardiac fibroblast differentiation via the miR-133b/CTGF axis; circSMAD4 134 promotes cardiac fibroblasts differentiation via the miR-671-5p/FGFR2 axis; circ-sh3rf3 135 inhibits cardiac fibroblasts differentiation via the GATA-4/miR-29a axis. In dermal fibrosis, circAMD1 136 promotes the differntiation of P63-mutated human dermal fibroblasts by targeting miR-27a-3p.

#### ncRNA regulates the immune microenvironment during fibrosis

In chronic obstructive pulmonary disease (COPD), miR-let-7 137 can regulate airway remodeling in the lungs by reducing IL-6 expression through direct targeting of IL-6 mRNA. miR-27b-3p derived from alveolar epithelial cells promotes the pro-inflammatory and anti-fibrotic effects of Flt3+ macrophages in PF 138. miR-302a-3p inhibits macrophage polarization towards M2 and suppresses bleomycin-induced pulmonary fibrosis 139. In non-alcoholic fatty liver disease (NALD), miRNA-rich exosomes secreted by immune cells have emerged as a new target for NAFLD therapy 140. In liver fibrosis, lncRNA Gm9866 promotes macrophage polarization for the development of liver fibrosis 139. During liver aging, T-reg-specific nuclear lncRNA Altre regulates optimal mitochondrial function as well as the T-reg-maintained hepatic immune microenvironment through the yin-yang 1 protein, thereby maintaining immunometabolic homeostasis in the aged liver 141. In myocardial fibrosis caused by myocardial infarction, Yu *et al.* reported a novel DC-expressed circRNA called circSnx5, which would inhibit DC maturation via miR-544/SOCS1; it also directly affects the nuclear translocation of PU.1, regulates the expression of downstream MHC class II, and modulates the function of DCs 142. In thyroid-related eye disease, miR-146a, miR-155, miR-96, and miR-183 were found to be involved in the immune response 143. In systemic lupus erythematosus, ncRNAs such as miR-146a, lnc-DC, miR-148a, circIBTK, circPOLR2A, etc. are involved in regulating signaling of innate immune signaling pathways, production of inflammatory cytokines, and activation of autoreactive T and B cells 144. In diabetic nephropathy-induced renal fibrosis, silencing of lncRNA Meg3 inhibited LPS-induced production of CCL-2 and CXCL-2 cytokines, which ameliorated renal fibrosis in mice 145.

#### ncRNA regulates fibrosis-associated cell autophagy, senescence

In PF, lncRNA MEG3 can inhibit NiO NPs-induced lung fibrosis by regulating autophagy in alveolar epithelial cells, which could be a potential therapeutic strategy for PF 146; circHECTD1 overexpression or HECTD1 knockdown reverses SiO2-induced autophagy in human primary lung fibroblasts and restores SiO2-induced fibroblasts through downstream autophagy activation, proliferation, and migration 147; CDKN2B-AS1 promotes lung fibroblast autophagy to alleviate the development of IPF through the miR-199a-5p/SESN2 axis 148. miR-34a, miR-29, and miR-200 families, as well as lncRNAs SIRT1-AS, TERRA, and MALAT1, can regulate alveolar epithelial/fibroblast senescence regulates the development of lung fibrosis 149. circRNA-kif26b mediates alveolar epithelial cell senescence through miR-346-3p and regulates microplastic-induced lung injury 150. In liver fibrosis, lncRNA NEAT1 enhances IGFBPrP1-induced autophagy in HSC via the miR-29b/Atg9a axis, thereby promoting the development of liver fibrosis 151; in alcoholic liver fibrosis, lncRNA XIST enhances alcohol-induced HSC autophagy via the miR-29b/HMGB1 axis 152; lncRNA PVT1 induces HSC autophagy under hypoxic conditions via miR-152/ATG14 153; in non-alcoholic cirrhosis, lncRNA MIR22HG inhibits autophagy to increase fibrosis via miRNA-9-3p/IGF1 axis 154. In renal fibrosis, lncRNA ENST00000453774.1 promotes reactive oxygen species defense and reduces the production of ECM-associated proteins involved in renal fibrosis by activating survival autophagy in proximal tubular epithelial cells of human renal tubules 155, and lncRNA SOX2OT promotes autophagy in tethered cells and attenuates fibrosis due to diabetic nephropathy 156; knockdown of lncRNA-TUG in renal tubular epithelial cells increased miR-223-3p expression and inhibited Klotho function aggravated renal tubular epithelial cell senescence 157. In the complication of uterine adhesions, which is mainly characterized by uterine fibrosis, Peng *et al.* established a circRNA-miRNA-mRNA regulatory network and found that hsa-circ-0047959, hsa-circ-0032438, hsa-circ-0047301, and miR-320c were all associated with endometrial cell autophagy 158. In myocardial fibrosis, lncRNA-H19 can drive cardiomyocyte senescence through the miR-19a/socs1/p53 axis 159.

#### ncRNA regulates DNA repair processes and cell cycle progression in fibrosis-associated cells

In PF, the exosomal miRNA Let-7 derived from menstrual blood-derived endometrial stem cells alleviates pulmonary fibrosis by regulating mitochondrial DNA damage 160. In myocardial fibrosis, miR-let-7d-3p directly targets HMGA2 to regulate cardiomyocyte DNA repair 161. In radiation-induced lung injury, overexpression of lncRNA LIRR1 in human bronchial epithelial cells leads to decreased expression of KU70, KU80, and RAD50 DNA repair proteins, resulting in DNA damage, and it also leads to decreased expression of cell cycle protein-dependent kinase 2, which regulates the cell cycle in human bronchial epithelial cells 162. In liver fibrosis, circ-0070963 inhibits the development of hepatic fibrogenesis by regulating the cell cycle of HSC through miR-223-3p/LEMD3 163. circBNC2 inhibits G2-M arrest in epithelial cells and prevents the occurrence of poor repair during fibrosis 164.

Some ncRNAs can simultaneously regulate several key processes of fibrogenesis, for example, miR-155 not only affects the JAK/STAT and TGF-β pathways, but also the number and function of neutrophils and macrophages, in addition, miR-155 also regulates epithelial-mesenchymal transition as well as the proliferation, migration and differentiation of fibroblasts, which promotes skin, myocardial, and liver fibrogenesis 165. Similarly, there can be multiple ncRNA expression changes in fibrosis of the same organ, such as in liver fibrosis where circPSD3, circCREBBP, miR-1291, circFBXW4, and miR-18b-3p expression are all altered, regulating HSC cell proliferation and differentiation as well as secretion of inflammatory factors and thus the progression of liver fibrosis 166. In myocardial fibrosis, the dysregulated expression of lncRNAs TDRG1, LINC01013, HOTAIR, miR-9, and miR-221/222 regulates the activation of ERK/JNK signaling pathway, the proliferation, migration, differentiation, and autophagy of cardiac fibroblasts as well as myocardial tissue inflammatory response which in turn promotes the progression of myocardial fibrosis 167, 168. The above findings suggest that ncRNAs play an important role in the development of fibrosis by regulating the key processes of fibrosis, and thus their role in fibrosis should not be ignored.

## Crosstalk of ubiquitin system and non-coding RNA in fibrosis

### Regulation of the ubiquitin system by ncRNAs

#### Inhibition of ubiquitinase function by microRNA binding to the 3'-UTR of ubiquitinase mRNAs

miRNAs directly degrade or inhibit target mRNAs by complete or incomplete pairing with the 3'-UTR of the target mRNA. In fibrotic diseases, miRNAs can inhibit the function of ubiquitinase by binding to the 3'-UTR of the ubiquitinase mRNA. LncRNAs and circRNAs can be used as ceRNAs to target miRNAs to regulate miRNA expression, to inhibit their binding to the 3'-UTR of target mRNAs, thereby regulating the progression of fibrotic diseases.

In renal fibrosis due to diabetic nephropathy, miR-27b-3p and miR-1228-3p bind to the ubiquitin-binding E2 enzyme variant (UBE2v1) 3'-UTR and inhibit UBE2v1-mediated lysine 63-linked ubiquitin chain formation 169. miR-154-5p binds to the Smurf1 3'-UTR and inhibits the Smurf1 binding to smad3, which subsequently regulates TGF-β1/smad3-mediated cell proliferation 170. circADAM9 targets miR-545-3p, which enhances the expression of the deubiquitinating enzyme USP15, activates the Keap1/Nrf2 pathway and leads to a more severe onset of fibrosis 171. In Aristolochic acid-induced renal fibrosis due to acute kidney injury, stable overexpression of miR-192 represses the expression of E3 ubiquitin ligase, murine double-minute 2 (a negative regulator of p53), which arrests the cell cycle in the G(2)/M phase, and this growth arrest promotes proximal renal tubular epithelial cell injury followed by fibrosis 172. In human and mouse obstructive nephropathy, let-7a-5p and miR-29-3p may target the E3 ubiquitin-protein ligase DTX4, leading to the development of renal fibrosis 173.

In PF, miR-15b can target the 3'-UTR region of Smurf2 mRNA and inhibit Smurf2 expression, the ubiquitination degradation pathway of heat shock protein 27 (HSP27) is blocked, and the degree of lung fibrosis is aggravated 174. LncRNA DLEU2 targets miR-369-3p and reduces the inhibitory effect of miR-369-3p on E3 ubiquitin ligase TRIM2, thereby promoting EMT in lung fibrosis and aggravating lung fibrosis 175. Yao *et al.* discovered that multiple dysregulated lncRNAs and miRNAs can regulate the expression of multiple ubiquitinases, such as TRIM39, TRIM32, and thus the progression of pulmonary fibrosis 176. Researchers also found that miR-125a-5p, derived from exosomes of macrophages from silicosis patients, inhibits Smurf1 expression, thereby mediating the differentiation of lung fibroblasts to myofibroblasts, which plays a key role in silicosis 177. In IPF, miR-424 reduces the protein expression of Smurf2, and then regulates myofibroblast differentiation during EMT, thereby accelerating fibrosis progression 118. lncRNA DLEU2 competitively binds to miRNA-369-3p, which attenuates its inhibitory effect on TRIM2, thereby promoting the fibrosis progression 178.

In liver fibrosis, miR-302c binds to the 3'-UTR of E6AP mRNA, and decreased expression of miR-302c leads to overexpression of E6AP, which attenuates hepatic fibrosis by inhibiting the TGF-β-induced MAPK signaling pathway 179. LncRNA H19 targets miR-148a, increases deubiquitinating enzyme USP4 expression, activates the TGF-β signaling pathway in hepatic stellate cells and hepatocytes, and exacerbates hepatic fibrosis 180. LncRNA GAS5 competitively binds to miR-28a-5p and increases the expression of the E3 ubiquitin ligase MARCH7, which mediates thermoproteins deposition, thereby preventing the development of NAFLD 181.

In dermal fibrosis, a bioinformatics study found that the expression of the E3 ubiquitin-protein ligase NEDD4L was regulated by 33 miRNAs 182. It has also been shown that miR-130a promotes collagen secretion, myofibroblast differentiation, and cell proliferation by targeting the deubiquitinating enzyme CYLD, decreasing CYLD expression, enhancing AKT activity, and leading to fibroplasia 183.

In myocardial fibrosis, has-circ-0000672 can act as a ceRNA, targeting miR-516a-5p and decreasing its expression, resulting in decreased binding of miR-516a-5p to its target gene, TRAF6 mRNA 3'-UTR, and facilitating the development of myocardial fibrosis 184. miR-181c targets the E3 ubiquitin ligase Parkin, Parkin expression is reduced, its ability to participate in autophagy, degradation of proteins, alteration of subcellular localization of proteins and protein-protein interactions is greatly reduced, and the degree of myocardial fibrosis is aggravated 185. miR-223 directly interacts with the FBXW7 mRNA 3'-UTR, reduces the expression of FBXW7, activates the AKT signaling pathway, and causes the heart to undergo physiological hypertrophy 186. miRNA-1 up-regulates the expression of UPS-associated mRNAs and affects the majority of the UPS components in the myocardium (19s, 20s, and E3 ligases), thereby leading to increased cardiomyocyte apoptosis, myocardial fibrosis, and myocardial remodeling 187. In the circRNA-miRNA-mRNA network constructed from bioinformatics analysis of tissue samples from patients with paroxysmal atrial fibrillation, circ-0054537, circ-0124644, circ-0113854, and circ-0082081 competitively bind to miR-766-3p, miR-513a-5p, miR-890, miR-1827, while miR-766-3p can target the genes of E2 ubiquitin-conjugating enzyme UBE2D and E3 ubiquitin ligase Cul5, miR-513a-5p targets E3 ubiquitin ligase FBXW7, E2 ubiquitin-conjugating enzyme UBE2D1, UBE2K genes, and miR-890 targets E2 ubiquitin-conjugating enzyme UBE2W, UBE2B and E3 ubiquitin ligase FANCL genes, and miR-1827 targets E3 ubiquitin ligase MDM2 and VHL genes, a component of the E3 ubiquitin ligase complex. More experimental studies are needed to demonstrate the regulation of ubiquitinases by ncRNAs in paroxysmal atrial fibrillation 188.

#### lncRNAs and circRNAs directly or indirectly physically bind to ubiquitinase and modulate ubiquitinase function

LncRNAs and circRNAs can directly bind to ubiquitinases and activate or silence ubiquitinase function, thereby regulating fibrotic disease progression. The binding of the lncRNA MaIL1 to Optineurin (OPTN, a ubiquitin adapter platform TBK1 kinase) stabilizes OPTN expression, enhancing OPTN aggregation, TBK1-dependent IRF3 phosphorylation, and type I interferon (IFN) gene transcription downstream of TLR4, regulating intrinsic immunity and influencing fibrosis progression 189. Heat shock proteins (HSP) are molecular chaperones that catalyze the correct folding of nascent and misfolded proteins. In renal fibrosis caused by diabetic nephropathy, lncRNA ENSMUST00000147869 directly binds to the heat shock protein HSPA9, reduces its expression through ubiquitination degradation, and inhibits the interaction between HSPA9 and HMGB1, thereby inhibiting the development of renal fibrosis 190. LncRNA CYP4B1-PS1-001 interacts with Nucleolin (NCL), disrupts the binding of ubiquitin ligase TRIM2 to NCL, and inhibits the ubiquitination and degradation of NCL from affecting the process of renal fibrosis 191. LncRNA ARAP1-AS2 directly binds to ARAP1, increases its ability to competitively bind to CIN85 with the E3 ubiquitin ligase Cbl, and decreases Cbl's ubiquitination of the epidermal growth factor receptor, thereby maintaining the sustained activation of the epidermal growth factor receptor, leading to exacerbation of high glucose-induced proximal tubular cell injury 192. In rat myocardial fibrosis, lncRNA MALAT1 binds to the enhancer of zeste homolog (EZH2) and promotes EZH2 activity in the nucleus of cardiomyocytes. Inhibition of ubiquitin-specific peptidase 22 (USP22) expression by EZH2 through H3K27me3 modification aggravates cardiac injury in SIMD rats 30.

#### lncRNA and circRNA are translated into proteins that regulate ubiquitinase function

Studies have shown that lncRNAs and circRNAs can be translated into small-molecule polypeptides in a variety of ways. Using PhyloCSF (a comparative genomics method that identifies evolutionarily conserved ORFs based on nucleotide sequence comparisons across multiple species), researchers have found that lncRNA INCR encodes TUBL, a protein with a ubiquitin-like (Ubl) structural domain, which plays a critical role at the protein level in the maintenance of homeostasis after skin injury by promoting the proliferation of keratinocytes 193.

### The ubiquitin system regulates ncRNA production, degradation

Ubiquitinases are not only regulated by ncRNA but also in turn regulate ncRNA production. E3 ubiquitination ligase APC (APCCdh1) mediates the degradation of Sen1, and a decrease in Sen1 leads to a decrease in ncRNA production 194. Ubiquitin-like modification of histone H2B and acetylation of AET3 inhibits ncRNA transcription from mRNA and reduces ncRNA production 195. LncRNA SINEUP molecule is transcribed from AS-Uchl1 (antisense ubiquitin carboxy-terminal esterase L1) gene in a head-to-toe arrangement with the target gene of the synonymous protein code, Uchl1. It can enhance the expression of target proteins in a specific and controllable manner without altering the level of target mRNAs 196. Using genome-wide CRISPR-Cas9 technology, Han *et al.* found that ubiquitin ligases may be involved in Cyrano-induced miR-7 degradation. It was demonstrated that ZSWIM8 (zinc finger SWIM-type containing 8) ubiquitin ligase recognizes the K493 lysine on the surface of AGO2, which in turn mediates the degradation of miR-7 by the proteasome. Deletion of the ZSWIM8 ubiquitin ligase results in the accumulation of a wide range of miRNAs in mammals, Drosophila, and nematodes. This suggests that ZSWIM8 ubiquitin ligase plays an important role in miRNA degradation 197. miRNA-1-1 and miRNA-1-2, derived from introns 2 and 12 of the E3 ubiquitin ligase MIB1 (Mindbomb Homogog 1), miRNA-1-1/miRNA-1-2 and MIB1 generate this abrogation 187. E3 ubiquitinating enzyme Smurf2 activation of cAMP-PKA-CREB signaling induces upregulation of miR-132 expression. miR-132 directly targets the 3'-UTR of CTGF, inhibiting CTGF production in hepatocytes and suppressing hepatic fibrosis 198. The E3 ubiquitin ligase Pellino1 promotes the activation of NF-κB and AP-1 which can promote the expression of miR-494-3p in cardiomyocytes (CFs). miR-494-3p induces CFs activation to promote myocardial fibrosis by inhibiting PTEN and amplifying phosphorylation of AKT, smad2/3, and ERK 199. It has also been shown that the ubiquitin system regulates the biosynthesis, bioprocessing processes, and metabolic processes of various types of RNAs 200. This also includes ncRNAs, such as the E3 ubiquitin ligase Roquin protein with a RING structural domain, which binds to miR-146a and reduces miR-146a in T cells by enhancing Dicer-mediated processing of pre-miR-146 as well as by inhibiting the half-life of mature miR-146a 201. Ago2 is the catalytic component of RISC that regulates miRNA expression, and it has been shown that AGO2 can be regulated by E3 ubiquitin ligase, which promotes its degradation and thus inhibits miRNA activity 202.

### ncRNAs synergize with the ubiquitin system to co-regulate key processes in fibrosis

**Figure 3:**
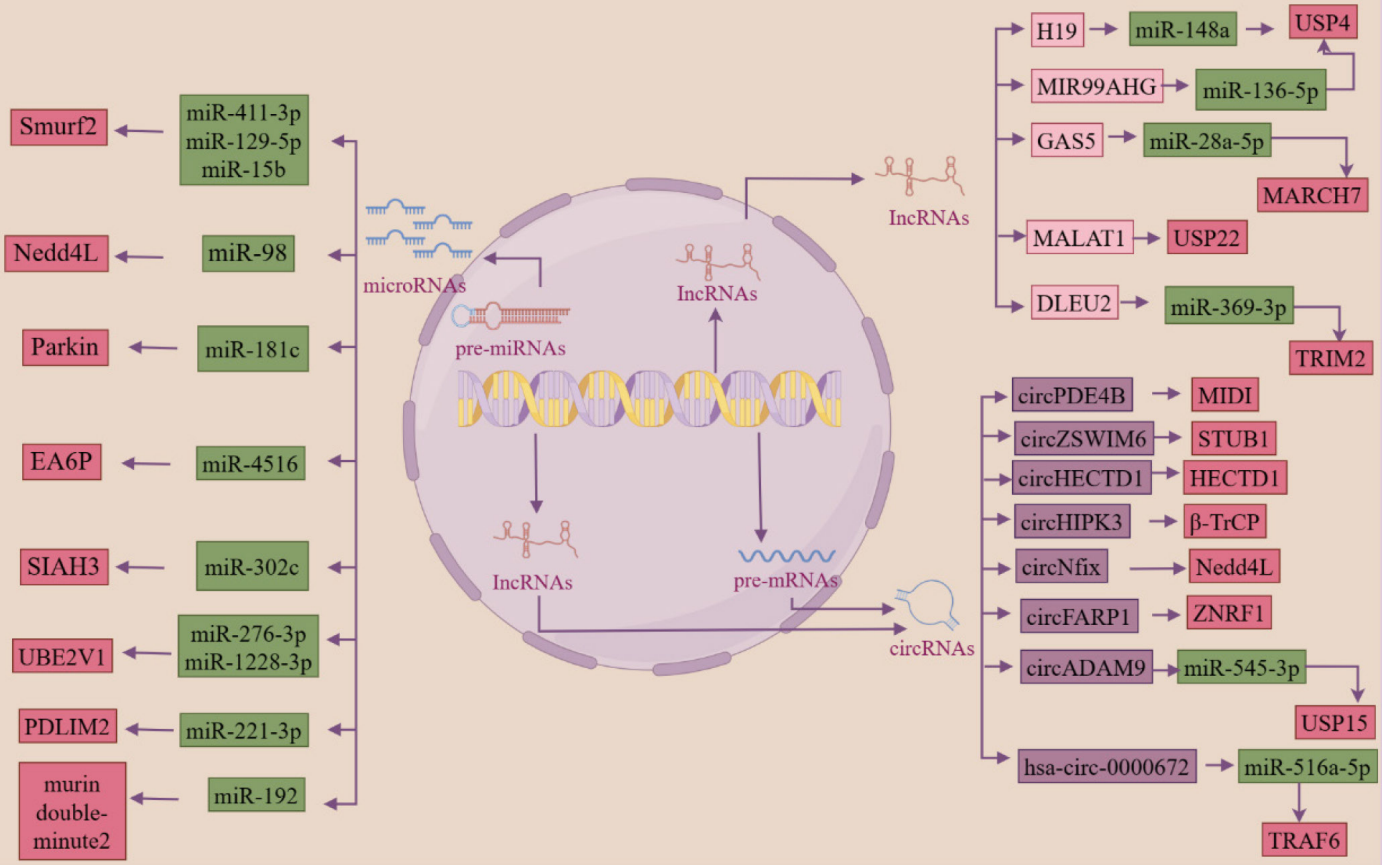
** ncRNA acts as an upstream regulator of the ubiquitin system, both of which work together to regulate the development of fibrosis (By FigDraw).** Regulatory RNAs include miRNAs, lncRNAs, and circRNAs, all three of which can be transcribed from linear RNAs, and circRNAs can also be transformed from lncRNAs. miRNAs and lncRNAs are single-stranded linear RNAs with a methyl guanosine cap (m7G) at the 5' end and a polyadenylate (poly) tail at the 3' end, while circRNAs are reverse spliced into a loop from linear RNAs that resist digestion by nucleic acid exonucleases. ' end polyadenylate (poly) tail, while circRNA is reverse spliced from linear RNA into a loop, which can resist digestion by nucleic acid exonucleases. Regulatory ncRNAs can act as upstream regulators of the ubiquitin system, which in turn regulates the progression of fibrosis. Green boxes are miRNAs; light pink boxes are lncRNAs; purple boxes are circRNAs; dark pink boxes are ubiquitin system-associated proteins.

Researchers have found that ncRNAs can regulate ubiquitinases or deubiquitinases in the ubiquitin system, which in turn are involved in the regulation of several key processes in the development of fibrosis. miR-411-3p 203, 204 targets Smurf2 to inhibit the activation of the TGF-β/smad signaling pathway and thereby inhibit bleomycin-induced dermal fibrosis and silica-induced pulmonary fibrosis. Reduction of miRNA-98 inhibits the TGF-β/smad2 pathway and attenuates renal fibrosis in diabetic nephropathy by increasing the expression of NEDD4L 205. miR-181c targets Parkin and smad7 in human cardiac fibroblasts and regulates the activation of the TGF-β/smad pathway and thus myocardial fibrosis 185. Smurf2 and miR-132 can co-regulate the expression of CTGF, a core mediator of liver fibrosis, to modulate cAMP-PKA-CREB signaling, which in turn regulates the progression of liver fibrosis 206. LncRNA H19 180 acts as a ceRNA to downregulate the expression of miR-148a and upregulate the expression of USP4, which stabilizes the TGFβRⅠ and activates the TGF-β/smad signaling pathway and promotes the development of liver fibrosis. In addition, miR-129-5p 207 was also targeted to Smurf2, promoting PTEN expression, ameliorating the impaired myocardial function caused by chronic heart failure, and inhibiting the development of fibrosis. circPDE4B can act as a scaffold to enhance the interaction between RIC8A and E3 ubiquitin ligase MIDI, ubiquitinate the degradation of RIC8A, inhibit the activation of the p38 signaling pathway, which in turn reduces the viability of chondrocytes and inhibits the catabolism of the extracellular matrix (ECM), preventing articular cartilage degeneration and promote repair 208. miR-15b targets the E3 ubiquitin ligase Smurf2, causing it to lose its function of ubiquitination degradation of HSP27, upregulating HSP27 expression and promoting cell migration, thus mediating the abnormally high migratory activity of fibroblasts in fibrotic lung tissues, which in turn promotes the onset and development of pulmonary fibrosis 174. circHECTD1 reduces HECTD1 expression in lung endothelial cells, leading to endothelial-mesenchymal transition, which promotes silica-induced pulmonary fibrosis 209. LncRNA MIR99AHG can act as a ceRNA to antagonize miR-136-5p-mediated down-regulation of USP4 expression, thereby deregulating miR-136-5p regulation of angiotensin-converting enzyme 2 (ACE2) expression and ultimately inhibiting the EMT process in lung fibrosis 210. LncRNA GAS5 binding to miR-28a-5p inhibited the binding of miR-28a-5p to the 3'-UTR of the E3 ligase MARCH7, resulting in the up-regulation of MARCH7 expression, which interacts with NOD-like receptor protein 3 (NLRP3) proteins, leading to the degradation of the NLRP3 proteasome, which inhibited the inflammation and the deposition of heat proteins, and attenuated the development of NAFLD 181. circZSWIM6 binds to the E3 ubiquitin ligase STUB1 binding site K125 on RPS14 (ribosomal protein S14) and inhibits RPS14 degradation to maintain the function of RPS14, regulate ECM metabolism and AMPK-related metabolism, and in turn, regulate chondrocyte senescence 211. miR-4516 targets the E3 ubiquitin ligase SIAH3, resulting in reduced aggregation of PINK1 to mitochondria and reduced mitochondrial autophagy, which in turn promotes the progression of renal fibrosis 212. circHIPK3 acts as a scaffold to enhance the interaction between the P21 mRNA, the binding protein HuR, and the E3 ubiquitin ligase β-Trcp interaction, leading to the ubiquitinated degradation of HuR, which in turn slows down cardiac aging 213. CircNfix inhibits myocardial regeneration in myocardial injury by enhancing the interaction of YBX1 (Y-box binding protein) with NEDD4L, which promotes the ubiquitinated degradation of YBX1 and inhibits the expression of cell cycle proteins A2 and B1 214. LncRNA MetBil up-regulates the expression of METTL3 through the ubiquitin-proteasome pathway, and METTL3-mediated modification of fibrosis-regulated genes by m6A modification regulates myocardial fibrosis occurrence 215. Knockdown of lncRNA DLEU2 inhibits IPF by regulating the microRNA-369-3p/TRIM2 axis 178. All the above studies indicate that ncRNA and ubiquitin system can act as two major regulators to jointly regulate the activation of fibrosis-related signaling pathways, the activation and differentiation of fibroblasts, mesenchymal stromal cells, the immune microenvironment, cellular autophagy, and the alteration of the cell cycle, etc. These studies are of great significance for the clinical prevention, diagnosis, and treatment of fibrosis (Figure 3).

## Summary and prospects

When various stimuli cause tissue damage, the tissue first undergoes a repair response through fibrin filling, cellular (fibroblasts, epithelial cells, endothelial cells, etc.) proliferation, and tissue remodeling; however, when the stimulus persists, the tissue repair response is dysfunctional, and if left unchecked, progresses to fibrosis. Fibrosis is responsible for up to 45% of deaths in industrialized countries. This shows that fibrosis has serious consequences for human life. Surprisingly, in recent years, preclinical models, and clinical trials in a variety of organ systems have shown that fibrosis is not an irreversible process, but rather a highly dynamic one. Human intervention may be able to reverse or even cure fibrosis. Studies by multiple investigators have identified the ubiquitin system as being involved in key processes in the development of fibrosis, and based on these studies, small molecule drugs targeting the ubiquitin system have been designed to treat fibrosis. However, with the deepening of the research, several researchers have found that ncRNAs have a powerful regulatory function that can regulate the expression of ubiquitinase and deubiquitinase, and thus regulate the progression of fibrosis. This suggests that on the one hand, the regulatory role of ncRNAs must be considered when targeting the ubiquitin system for the prevention, diagnosis, and treatment of fibrosis, otherwise, it may be counterproductive; on the other hand, the immunogenicity of ncRNAs is weaker than that of macromolecular proteins, especially circRNAs, which have obvious superiority when serving as a therapeutic target for fibrosis, and if the upstream ncRNAs can modulate the expression of the ubiquitin system, then ncRNAs can be targeted, and ncRNAs will be used as a therapeutic target. ncRNA as a target, its safety is higher than that of protein target to some extent; or the combination of the two can play a more effective therapeutic role. Undeniably, ncRNA as a therapeutic agent itself has some inherent disadvantages, such as too many effector targets, which may lead to systemic effects. Secondly, how to deliver ncRNA therapeutic agents to the site of injury and how to maintain their stability still deserves deep thinking, and many delivery vectors such as viral vectors, liposomes, and polymers have been developed, but these vectors still need to be further optimized and tested 216. Therefore, an in-depth investigation of how the ncRNA and ubiquitin systems work together to regulate the progression of fibrosis is of great clinical importance in the search for fibrosis prevention, diagnosis, and therapeutic targets.

## Figures and Tables

**Figure 2:**
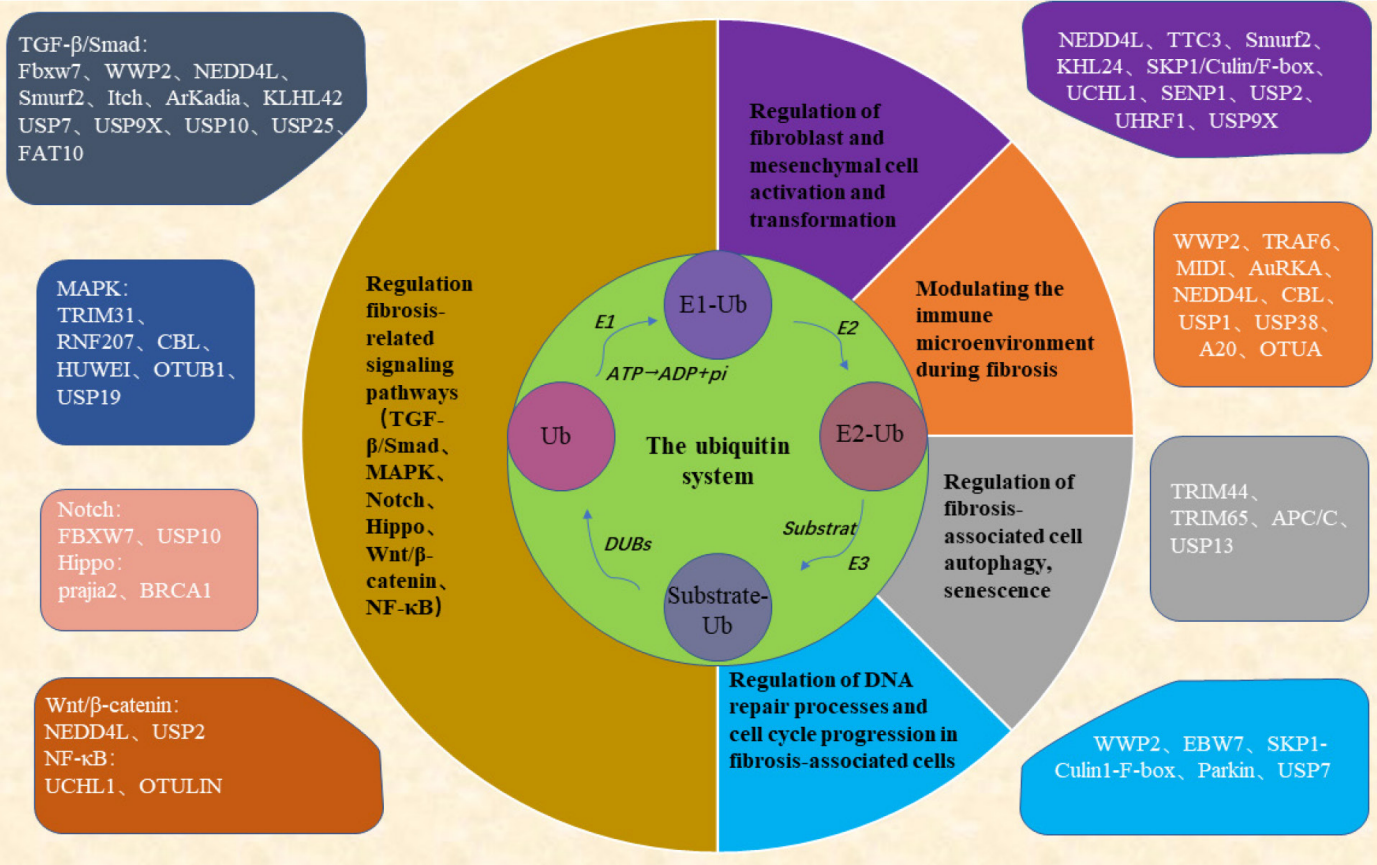
** The ubiquitin system regulates key processes in the development of fibrogenesis.** Ubiquitin proteins ubiquitinate substrate proteins in the presence of EI, E2, and E3 enzymes, a process that can be reversed by the deubiquitinating enzymes DUBs. The ubiquitin system can be involved in key events in fibrosis regulating the progression of fibrosis. Fbxw7, WWP2, NEDD4L, Smurf2, Itch, ArKadia, KLHL42, USP7, USP9X, USP10, USP25, FAT10, TRIM31, RNF207, CBL, HUWEI, OTUB1, USP19, FBXW7, USP10, prajia2, BRCA1, NEDD4L, USP2, UCHL1, OTULIN can regulate the activation of fibrosis-related signaling pathways; NEDD4L, TTC3, Smurf2, KHL24, SKP1/Culin/F-box, UCHL1, SENP1, USP2, UHRF1, USP9X can regulate the activation and differentiation of fibroblasts and mesenchymal cells; WWP2, TRAF6, MIDI, AuRKA, NEDD4L, CBL, USP1, USP38, A20, OTUA can regulate the immune microenvironment of fibrotic tissues; and TRIM44, TRIM65, APC/C, USP13 can regulate autophagy and senescence in fibrosis-associated cells; and WWP2, EBW7, SKP1-Culin1-F-box, Parkin, USP7 can regulate cell cycle and DNA damage in fibrosis-associated cells.

**Table 1:** Common ubiquitin enzymes and their functions

Ubiquitinase	Function	Common Types
Ubiquitin-activating enzyme, E1	Contain a conserved cysteine residue at a fixed position in its structure, which activates ubiquitin through the formation of a high-energy thioester bond between the cysteine residue and the C-terminus of ubiquitin.	Typical E1 enzymes: UBA1, UBA6, UBA7, SAE, NAE
		Atypical E1 enzyme: UBA4, UBA5, ATG7
Ubiquitin-conjugating enzymes, E2	1. Contains a conserved core structural domain of about 150 amino acid residues 2. the center of the structural domain is a cysteine residue that determines its enzymatic activity. 3. E1 and E3 are connected to E2 by the same motifs, so E2 must shuttle back and forth between E1 and E3 during the reaction cycle.	UBE2A, UBE2B, UBE2C, UBE2D, UBE2E, UBE2F, UBE2G, UBE2H, UBE2I, UBE2J, UBE2K, UBE2L, UBE2M, UBE2N, UBE2O, UBE2P, UBE2Q, UBE2R, UBE2S, UBE2U, UBE2V, UBE2W, UBE2AZ, BIRC6
Ubiquitin-protein ligases, E3	Linking ubiquitin-conjugating enzymes and specific substrates, transferring activated ubiquitin chains to lysine residues of specific substrates, and purposeful degradation of proteins by recognizing multimeric ubiquitin chains	HECT structure: E6AP, Nedd4, Smurf1/2, Rsp5, Hyd, Ure-B1
		RING structure: CBL, Mdm2, IAP, parkin, Ubr1, Rbx1, APC/Cyclosome, SCF type, TRIM, Arkadia
		U-box structure: UFD2a/b, CHIP, UIP5, CYC4
Deubiquitinating enzyme (DUBs)	1. Ubiquitin is released from substrate proteolysis 2. Catabolism of ubiquitination inhibitors 3. Recycling of ubiquitin molecules 4. Proofreading the ubiquitination process	USP: USP22, USP12, USP9X and so on
		SENP: SENP1, 2, 3, 5, 6 and so on
		JAMM: MYSM1, PRPF8, EIF3S3 and so on
		OUT: OTUB1/2, OTULIN, A20 and so on
		MJD: JOSD1/2, ATXN3L, ATXN3
		MINDY: MINDY1/2/3/4
		UCHL: UCHL1/3/5, BAP1

**Table 2:** Involvement of the ubiquitin system in regulating the development of fibrosis

Ubiquitinating/de- ubiquitinating enzyme	Effect	Expression	Disease	Reference
USP22	Promote	Up-regulate	Cardiac fibrosis	29, 30
USP13	Inhibit	Down-regulate	Lung fibrosis	31, 32
A20	Inhibit	Down-regulate	Lung fibrosis	33-35
FAT10	Promote	Up-regulate	Renal /Cardiac/liver fibrosis	36-38
UCHL1	Promote	Up-regulate	Cardiac fibrosis	39-41
Smurf1	Promote	Up-regulate	Ligamentum Flavum fibrosis	42, 43
WWP2	Promote	Up-regulate	Cardiac fibrosis	44-46
NEDD4L	Inhibit	Down-regulate	Pulmonary fibrosis	47-50
Itch	Inhibit	Up-regulate	Marrow/Cardiac fibrosis	51, 52
FBXW7	Inhibit	Down-regulate	Lung/Liver fibrosis	53, 54
TRIM31	Inhibit	Down-regulate	Hypertensive renal disease	25, 55
